# AR signaling context and the apparent lack of synergy between AR-targeted therapy and PARP inhibition

**DOI:** 10.1073/pnas.2611970123

**Published:** 2026-10-30

**Authors:** Susu Zhou, Che-Kai Tsao

**Affiliations:** ^a^ Division of Hematology and Oncology, Department of Medicine, State University of New York Upstate Medical University, Syracuse, NY 13210; ^b^ Division of Solid Tumor Medical Oncology, Department of Medicine, Northwell Health Cancer Institute, New York, NY 11042

We read with great interest the recent article by Traphagen et al. (2026 Jan 27) (1) reporting a lack of synergy between androgen receptor (AR)–targeted therapies and poly(ADP-ribose) polymerase (PARP) inhibitors in homologous recombination (HR)–proficient prostate cancer. This study provides important insights into the role of AR signaling in PARP inhibitor sensitivity; however, several aspects warrant further consideration.

In their castration-sensitive models, androgen deprivation (AD) alone markedly reduced baseline proliferation to approximately 15% of control levels (figure 1A, bar graph). Critically, this profound suppression of baseline viability appears to result in a “pseudoresistance” phenotype in the dose–response curves, where the IC_50_ values for PARP inhibitors are paradoxically increased under AD conditions compared to dihydrotestosterone (DHT)-proliferating conditions. Such a low baseline proliferation creates a floor effect that narrows the dynamic range, making it mathematically and biologically difficult to detect any synergistic growth inhibition. Therefore, the observed lack of synergy may, at least in part, partly reflect experimental limitations rather than true independence from AR signaling.

In their castration-resistant models with pharmacological AR inhibition, cell growth appears comparable with or without enzalutamide (figure 1C, bar graph), indicating that the cell lines used (22Rv1, LNCaP-abi, and LN95) are inherently insensitive or resistant to enzalutamide. While second-generation androgen receptor pathway inhibitors (ARPIs) remain clinically effective for many patients with castration-resistant disease, these specific in vitro models demonstrated no significant growth inhibition even at a high concentration (10 µM) of enzalutamide. This is consistent with prior reports showing these cells exhibit limited responsiveness to enzalutamide or other forms of AR signaling inhibition (2). As noted by the authors, 22Rv1 cells express AR-V7, a splice variant circumventing the need for the AR ligand-binding domain targeted by enzalutamide (3). Consequently, since enzalutamide fails to meaningfully modulate the AR-mediated transcriptional program, including DNA repair regulation—the proposed mechanism underlying sensitization to PARP inhibition—synergy is unlikely to be observed. Therefore, the absence of synergy in these models may reflect intrinsic enzalutamide resistance rather than a true lack of pathway interaction.

While clinical evidence is mixed, efficacy in HR-proficient prostate cancer may not be uniformly negligible. Although the MAGNITUDE trial showed no significant benefit from adding niraparib to abiraterone/prednisone in patients without HR alterations (4), recently meta-analyses have shown that combining PARP inhibitors with AR-targeted therapies significantly improves progression-free survival compared with AR-targeted therapy alone, regardless of HR status (5, 6). Furthermore, alterations in DNA repair–related genes that do not cause classical HR defects, such as CDK12 or LIG1, may also sensitize tumors to PARP inhibitors through alternative non-HR mechanisms (7, 8). Thus, further investigation using models that retain ARPI responsiveness or harbor specific non-HR DNA repair alterations is essential to better define the biological contexts of potential synergy.

Nevertheless, we appreciate the authors’ efforts in exploring this important and clinically relevant question. Their finding that PARP1-selective inhibition is noninferior to dual PARP1/2 inhibition in modulating AR-driven transcription may have important therapeutic implications, facilitating the development of PARP1-specific agents that retain biological activity while potentially mitigating myelosuppression.

## Data Availability

There are no data underlying this work.
